# Are Parents the Key? How Parental Suicide Stigma and Suicide Literacy Affect Help-Seeking Attitudes and Intentions for their Child

**DOI:** 10.1007/s10964-023-01841-3

**Published:** 2023-08-17

**Authors:** Colette T. Burke, Alison L. Calear, Tegan Cruwys, Philip J. Batterham

**Affiliations:** 1 School of Medicine and Psychology, The Australian National University, Building 39 Science Road, Canberra, ACT 2601 Australia; 2 Centre for Mental Health Research, The Australian National University, 63 Eggleston Road, Acton, ACT 2601 Australia

**Keywords:** Suicide, Stigma, Literacy, Help-seeking, Parent, Adolescent

## Abstract

Suicide is the leading cause of death among Australian young people, yet rates of help-seeking for suicidal ideation and behaviors in this population are concerningly low. In this study, the relationships between parental suicide stigma, parental suicide literacy, and their attitudes and intentions toward seeking professional help for their child if they were to express thoughts of suicide are investigated. Understanding this influence is critical given parents are key facilitators of their child’s access to and engagement with professional mental health services. An online survey was administered to 302 parents of children aged-12–18 (M_age_ = 45.36, SD_age_ = 6.23; 91.4% female). Parental suicide stigma was significantly associated with more negative help-seeking attitudes and lower help-seeking intentions. Other significant predictors of more positive help-seeking attitudes included parental self-efficacy and having a child with no history of suicidal ideation. Higher help-seeking intentions were associated with female gender, living in an urban area, and positive help-seeking attitudes. Parental suicide literacy was not significantly associated with help-seeking. Practically, outcomes of this study may inform the development and implementation of targeted education programs to increase parental help-seeking for their children.

## Introduction

Suicide is a devastating global phenomenon with significant public health implications. Of particular concern are the high rates of suicide among young people. In Australia, suicide is the leading cause of death among those aged 15 to 24 years, and the second leading cause for those aged 1 to 14 years (Australian Bureau of Statistics, 2021). Thus, adolescence constitutes a critical period of elevated risk for deaths by suicide. The promotion of help-seeking in adolescents at risk of suicide is paramount, since earlier access to support and assistance is associated with improved outcomes and reduced distress (Cosgrave et al., 2007). Yet rates of professional help-seeking by young people experiencing suicidal ideation and behaviors are critically low (Luoma et al., 2002; Wu et al., 2010). Given parents are one of the primary sources of emotional support for adolescents and regularly facilitate their access to professional mental health services (Sayal, 2005; Zwaanswijk et al., 2003), it is particularly important to establish the factors that help or hinder parents’ likelihood of seeking professional help for their child. This study examined several of those factors, which may influence parental attitudes and intentions toward seeking help for adolescents at risk of suicide.

### Adolescent Suicide and Help-Seeking

Data suggests that, following puberty, rates of suicide increase with age until stabilizing in young adulthood (Spirito & Esposito-Smythers, 2006). This increase may be driven by multiple factors, including the greater likelihood of psychosocial stress and negative life events during adolescence, such as interpersonal difficulties, bullying, and concerns around gender identity and sexual orientation (King & Merchant, 2008); the onset of more psychiatric and substance-use disorders than in childhood (Shaffer et al., 1996); a shift in circadian rhythm that may lead to sleep disturbances (McGlinchey et al., 2017); and a greater access and vulnerability to online and social media influences (Marchant et al., 2017).

Many individuals who attempt or die by suicide never come into contact with health services (Luoma et al., 2002). In a national survey in the United States, less than half of the adolescents that had attempted suicide in the preceding year reported they had engaged with mental health services in that time (Wu et al., 2010). Parents are one of the primary sources of emotional support for children and adolescents. They often hold critical roles in identifying when a problem exists, facilitating access to mental health care (for example, by referring, transporting and paying for their child to access the service), and actively participating in effecting change – by communicating with the mental health professionals and maintaining a climate within which their child can develop cognitively, emotionally, and socially (Villatoro et al., 2018). Therefore, it is particularly important to establish the factors that help or hinder parents’ likelihood of seeking professional help for their child. By identifying these barriers and facilitators, it may then be possible to identify ways to improve rates of help-seeking and access to services in young people.

### Factors Associated with Help-Seeking

Little is known about why those at risk of suicide do not seek help. By contrast, extensive research has been conducted to explain the low rates of help-seeking among those with broader mental health problems. Two potentially modifiable barriers to help-seeking that have been identified in previous research are mental health literacy and stigma. The term *mental health literacy* refers to “knowledge and beliefs about mental disorders which aid their recognition, management or prevention” (Jorm et al., 1997, p. 182). It includes: the ability to recognize specific disorders; seek mental health information; knowledge of risk factors, causes, self-help interventions and professional help available; and attitudes that facilitate appropriate help-seeking (Jorm et al., 1997). *Mental health stigma* refers to negative attitudes, judgements or stereotypes toward individuals with any form of mental illness, which may lead to devaluation, exclusion and discrimination (Barney et al., 2006). Existing research has found that low mental health literacy and high mental health stigma are associated with a reluctance to accept help from mental health professionals, lack of compliance with treatment recommendations, and pattern of inappropriate and inconsistent service use (Barney et al., 2006; Bonabi et al., 2016). Similar patterns seem to exist in the relationships between *suicide* literacy, stigma, and help-seeking. A recent community-based study demonstrated that, as is true for mental health generally, low suicide literacy and high suicide stigma were associated with more negative attitudes toward help-seeking and lower intentions of seeking help for suicidal ideation (Calear et al., 2014b).

While there is growing evidence linking literacy and stigma to *one’s own* help-seeking attitudes and intentions, there is very little research assessing the impact of these factors on help-seeking for other people’s mental health or suicidal ideation and behaviors – including a parent’s for their child. Some of the most common reasons cited by parents for not seeking help for their child’s mental health are: being unsure whether their child needed help, not knowing where to get help, or thinking the problem would get better by itself (Lawrence et al., 2015). Another study found that parental help-seeking was influenced by their perception of the problem, perceived ability to cope, and knowledge of child mental health problems and services (Sayal et al., 2010). Each of these issues is broadly related to low levels of knowledge about childhood mental health disorders – that is, low mental health literacy.

Parental stigma also seems to be predictive of help-seeking for both childhood externalizing disorders (Dempster et al., 2013) and mental health difficulties more generally (Sayal et al., 2010). Here, the barriers cited were feelings of embarrassment, blame or stigma, concerns about them or their child being labelled, the potential adverse consequences of their child receiving a diagnosis, and, most extremely, fears that their child would be removed from their care (Sayal et al., 2010). Another study focused on adolescent depression found that a parent’s depression stigma and literacy played an important role in the way they responded to adolescents when they were depressed, and that improving these two areas would likely lead to more adaptive parental responses (Johnco & Rapee, 2018). But again, less is known about parental help-seeking for a child with suicidal ideation or behaviors. Understanding the influence of parental knowledge of and attitudes toward suicide is an initial step in this process and, to our knowledge, one that has not yet been explored in the literature.

Other factors that have been identified as associated with help-seeking for mental health problems and suicidality include: self-efficacy, exposure to poor mental health or suicidal ideation and behaviors, positive past help-seeking experiences, and various demographic factors. Self-efficacy refers to a person’s beliefs about their capacity to utilize personal resources to take control of their life course (Bandura, 1982). Research shows people are more likely to engage in a behavior if they believe their efforts will be successful (Bandura, 1982, 2006). Thus, negative appraisal of one’s ability to access treatment to improve their mental health (that is, low self-efficacy for seeking mental healthcare) undermines appropriate help-seeking (Moore et al., 2015). One’s help-seeking may also be influenced by their history of exposure to suicide. Some studies have found no significant relationship in this respect (Calear et al., 2014b), while others have found that exposure to suicide leads to more negative attitudes toward help-seeking (Chan et al., 2014). Having a child with more severe or persistent problems has been shown to increase the likelihood of a family accessing treatment (Merikangas et al., 2011), as has a parent receiving mental health care themselves or having positive past experiences with help-seeking (Zwaanswijk et al., 2003). Demographic factors such as older age and female gender (Calear et al., 2014b), urban dwelling (Cohen & Hesselbart, 1993), not being of an ethnic minority (Barker & Adelman, 1994), and higher socioeconomic status (Pumariega et al., 1998) have all been identified as being significantly associated with help-seeking.

## Current Study

Given the critical gatekeeper role that parents often hold in facilitating their child’s access to mental health support services, there is a need to identify potentially modifiable factors that promote or prevent parental help-seeking for an adolescent’s suicidal ideation. The aim of the current study was to examine the relationships between parental suicide literacy, parental suicide stigma, and their attitudes and intentions toward seeking professional help for their adolescent child if they were to express thoughts of suicide. These aims were tested after adjusting for a range of demographic and psychosocial factors that may confound or be associated with the relationships, including self-efficacy, exposure to suicide, a child’s history of service use, emotional and behavioral problems and suicidal ideation, and parent age, gender, remoteness, socio-economic advantage, and level of education. It was hypothesized that higher parental suicide literacy and lower parental suicide stigma would be associated with more positive help-seeking attitudes and higher intentions of seeking help for a child expressing suicidal ideation.

## Methods

### Participants & Procedure

Approval to conduct this study was obtained from the Human Research Ethics Committee of the Australian National University (Protocol 2019/295). A cross-sectional online survey was administered to individuals residing in Australia who self-identified as the parent of an adolescent aged between 12 and 18 years – a period of increased risk for suicidal ideation and behaviors. Participants were recruited by two means: via secondary school newsletters in the Australian Capital Territory and targeted *Facebook* advertising. When compared to postal or telephone sampling, online sampling has been identified as more successful in recruiting hard-to-reach populations, and as more efficient, flexible and cost-effective (Batterham, 2014). Online samples are also typically comparable to traditional samples in respect to their representation of age, gender, education and cultural diversity (Batterham, 2014).

Through these two means, a total sample of 302 Australian parents was recruited. Table 1 shows the demographic characteristics of this sample. The sample was predominantly female (91%), highly educated (89% had a tertiary education), and, similarly to the Australian population, primarily located in urban areas (71%). The proportion of parents with a child with a history of suicidal ideation was higher (48%) than the prevalence of that in the adolescent population (29.9%; Evans et al., 2005).

**Table 1:** Demographic Characteristics of the Sample

	*N*	Frequency or *M*	% or SD
Age*	302	45.36	6.23
Female	302	276	91.4%
State/Territory			
Australian Capital Territory (ACT)	299	84	27.8%
New South Wales (NSW)	299	70	23.2%
Northern Territory (NT)	299	5	1.7%
Queensland (QLD)	299	56	18.5%
South Australia (SA)	299	19	6.3%
Tasmania (TAS)	299	8	2.6%
Victoria (VIC)	299	42	13.9%
Western Australia (WA)	299	15	5%
ASGS Remoteness Area			
1: Major cities of Australia	299	213	70.5%
2: Inner regional Australia	299	64	21.2%
3: Outer regional Australia	299	20	6.6%
4: Remote Australia	299	1	0.3%
5: Very remote Australia	299	1	0.3%
Index of Relative Socio-Economic Advantage (10) and Disadvantage (1) (IRSAD)	299	7.06	2.77
English as an additional language	302	25	8.3%
Aboriginal or Torres Strait Islander	302	5	1.7%
Number of children	302	2.29	1.01
Living arrangements			
Both parents in the same home	302	196	64.9%
Both parents in separate homes	302	35	11.6%
One parent only	302	66	21.9%
Neither parent, foster/kinship carer	302	5	1.7%
Marital status			
Single	302	17	5.6%
Married or domestic partnership	302	217	71.9%
Widowed	302	3	1%
Divorced	302	39	12.9%
Separated	302	26	8.6%
Education level			
Did not complete high school	302	8	2.6%
Completed high school	302	26	8.6%
Certificate/diploma/associate degree	302	102	33.8%
Bachelor’s degree	302	85	28.1%
Higher degree	302	81	26.8%

*N* Number of responses, *M* Mean, *SD* Standard deviation

*Participants’ ages ranged from 24- to 67-years-old

In both recruitment platforms, participants were invited to complete a survey titled “Supporting the Mental Health of Your Teen”, which took an average of 18 minutes. Direct references to suicidality were omitted from the title to neither attract nor discourage respondents. Participants needed to provide informed consent and confirm parental status (“Do you have one or more children [either biological or non-biological] between the ages of 12 and 18?”) before commencing the survey. They were provided with a list of help-seeking resources at its conclusion. Participants were not compensated for completing the survey.

### Measures

#### Suicide stigma

Parental suicide stigma was measured using the short form of the Stigma of Suicide Scale (SOSS-SF; Batterham et al., 2013). The 16-item SOSS-SF assesses stigmatizing attitudes of general community members toward those who die by suicide. Each item consists of a one- or two-word descriptor that may be applied to a person who dies by suicide, such as “pathetic”, “lonely” or “brave” (Batterham et al., 2013). Participants rate how much they agree with each item on a five-point Likert scale ranging from “Strongly Disagree” (1) to “Strongly Agree” (5), where higher scores indicate higher levels of stigma toward those who die by suicide (Batterham et al., 2013). The SOSS-SF has three subscales – the primary subscale assesses stigma (eight items; e.g., “pathetic”), the second, attribution of suicide to isolation or depression (four items; e.g., “lonely”), and the third, normalization or glorification of suicide (four items; e.g., “brave”) (Batterham et al., 2013). In the current study, all three of these subscales were included and analyzed separately. The subscales obtained similar to reported levels of internal consistency – for the stigma subscale, ɑ = 0.84; for the isolation/depression subscale, ɑ = 0.72; and for the glorification/normalization subscale, ɑ = 0.79.

#### Suicide literacy

Parental suicide literacy was measured using the short form of the Literacy of Suicide Scale (LOSS-SF; Calear et al., 2014a). The 12-item LOSS-SF assesses knowledge of suicide in four domains – signs and symptoms, causes or the nature of suicidality, risk factors, and treatment and prevention (Calear et al., 2014a). Each item is answered as “True”, “False” or “I Don’t Know” (Calear et al., 2014a). Correct item responses are allocated a score of 1, and incorrect (or “I Don’t Know”) responses a score of 0. Total scores range from 0 to 12, where higher scores indicate higher levels of suicide literacy (Calear et al., 2014a). Given this measure is an assessment of knowledge, with correct and incorrect responses, it is not appropriate to report traditional psychometric properties like internal consistency.

#### Help-seeking attitudes and intentions

Parental *attitudes* toward help-seeking and *intentions* to seek professional help from six sources were assessed. Both measures were adapted to ask about help-seeking for one’s child, rather than oneself, and to be answered in response to a situational vignette based on similar past studies (e.g., Burns & Rapee, 2006; Jorm et al., 2006), which read as follows: *“Imagine the following hypothetical situation… Your child comes to you and tells you they are feeling so awful that life is not worth living, and that they are thinking of ending their life.”* As above, direct references to suicidality and more specific terminology were omitted to neither attract nor discourage responses, and so that participants did not require suicide literacy to understand the vignette.

The help-seeking *attitudes* measure was an adapted version of the updated 10-item short form of the Attitudes Toward Seeking Professional Psychological Help Scale (ATSPPHS-SF; Calear et al., 2014b; Fischer & Farina, 1995). Items (such as “I would want to get professional help if this situation continued for a long period of time”) are rated on a four-point Likert scale ranging from “Disagree” (0) to “Agree” (3) (Fischer & Farina, 1995). Total scores range from 0 to 30, where higher scores indicate more positive parental attitudes toward seeking professional psychological help for their child. Having modified the scale, the level of internal consistency was lower in this study (ɑ = 0.67), compared to the original (ɑ = 0.85; Fischer & Farina, 1995). The removal of items was examined as a potential solution to this suboptimal internal consistency. Removing Items 4, 5, and 6 produced the highest level of internal consistency (ɑ = 0.70), however the results of all analyses remained unchanged when using this alternate version. Thus, the full scale was retained.

The help-seeking *intentions* measure was modelled on the General Help-Seeking Questionnaire (GHSQ; Wilson et al., 2005). In the development and validation of the GHSQ, the authors found significant associations between participants’ help-seeking intentions and them actually seeking help from the corresponding source in the following three weeks (Wilson et al., 2005). It assessed intentions to seek help from six professional sources – (a) general practitioner or doctor, (b) school counsellor or nurse, (c) clinical psychologist or mental health professional, (d) psychiatrist, (e) crisis support service, and (f) emergency department; or, (g) none of those. Items are rated on a seven-point Likert scale ranging from “Extremely Unlikely” (0) to “Extremely Likely” (6), where higher scores indicate higher parental intentions to seek professional help for their child (Wilson et al., 2005). For analyses, help-seeking intentions were averaged as a combined score, ranging from 0 to 36. Given the GHSQ is more of an index than a scale (with individual items not expected to correlate highly), a unidimensional structure cannot be assumed and internal consistency was not evaluated.

#### Self-efficacy

Parental *self-efficacy* was assessed using the nine-item Self-Efficacy in Seeking Mental Health Care Scale (SE-SMHC; Moore et al., 2015), again adapted to ask about help-seeking for one’s child, rather than oneself, and to be answered in response to the situational vignette above. This scale measures one’s confidence in knowing how to access mental health care and communicate with staff (SE-Knowledge subscale; five items), as well as how to cope with the social and interpersonal consequences of seeking care (SE-Coping subscale; four items) (Moore et al. 2015). Items are rated on a 10-point Likert scale ranging from “No Confidence” (1) to “Complete Confidence” (10) (Moore et al., 2015), where higher scores indicate greater parental self-efficacy for seeking professional mental health care for their child. Scores for SE-Knowledge (ranging from 5 to 50), SE-Coping (ranging from 4 to 40), and SE-Total (ranging from 9 to 90) were all included and analyzed separately. Having modified the scale, the level of internal consistency was acceptable in this study (for SE-Knowledge, ɑ = 0.80; for SE-Coping, ɑ = 0.76; for SE-Total, ɑ = 0.85), compared to the original (for SE-Knowledge, ɑ = 0.87; for SE-Coping, ɑ = 0.87; for SE-Total, ɑ = 0.93) (Moore et al., 2015).

#### Exposure to suicide

*Exposure to suicide* was assessed by a 10-level multiple choice item (consistent with Calear and colleagues (2014b)) – from “I have had no exposure to suicide” (0) to “I have attempted suicide myself” (9). Each participant was assigned a score between 0 and 9, based on their highest level of exposure.

#### Child’s history of emotional or behavioral problems and service use

Three “Yes/No” questions gauged a participant’s *child’s history of emotional or behavioral problems* and *service use* over the preceding twelve months (consistent with Johnson and colleagues (2016)) – (a) “Have any of your children been seen by a health service provider for emotional or behavioral problems in the past 12 months?” (if answered “Yes”, followed by “Which of the following health service providers did your child/children use?” including options for general practitioner/doctor, school counsellor/nurse, clinical psychologist/mental health professional, psychiatrist, crisis support service, emergency department, or other); (b) “Have any of your children been given a formal mental health diagnosis in the past?” (if answered “Yes”, followed by “Which diagnosis did your child/children receive?” including options for anxiety disorder, mood disorder, personality disorder, attention-deficit/hyperactivity disorder/oppositional defiance disorder/conduct disorder, autism spectrum disorder, eating disorder, substance abuse disorder, schizophrenia/psychosis, or other); and finally, (c) “Have any of your children ever expressed thoughts of suicide?”.

#### Demographics

Participants were asked to provide their age, gender, linguistic diversity, Aboriginal or Torres Strait Islander identification, postcode, marital status, level of education, living arrangements, and number of children living in their household. Postcode was used to calculate an Index of Relative Socio-economic Advantage and Disadvantage (IRSAD) (Australian Bureau of Statistics, 2018b) – from 1 (most disadvantaged) to 10 (most advantaged); and Australian Statistical Geography Standard (ASGS) Remoteness Area (Australian Bureau of Statistics, 2018a) – from 1 (major cities) to 5 (very remote).

### Analyses

Prior to analyses, data were screened for accuracy, missing cases, outliers, and fit to the assumptions of hierarchical multiple regression. Altogether, 407 participants clicked on and started the survey but 105 were removed – 29 did not qualify (they were not the parent of a child aged between 12 and 18), 56 failed to complete the survey, and 20 failed at least one attention check. Hence, the final sample consisted of 302 participants. At this point, there remained eight participants with missing data across 24 items, which were imputed using Expectation Maximization – a simple approach to imputing data, provided the number of cases was low and preliminary analyses indicated they were missing at random (Tabachnick & Fidell, 2013).

Principle Axis Factoring indicated the six GHSQ items had a single underlying “help-seeking intentions” factor, which accounted for 40.6% of the total variance and was the only factor to meet Kaiser (1960)’s criterion of having an eigenvalue greater than 1 (*eigenvalue* = 2.44). All the items were well-accounted for by this solution and loaded satisfactorily onto it. Thus, all items were retained and summed to form a total score.

Two hierarchical regression analyses were conducted to assess the associations between parental suicide stigma, parental suicide literacy, and help-seeking attitudes and intentions. Those variables found to be significant predictors of help-seeking in previous empirical research were controlled for in the models. In the first stage of the regression, demographic variables (age, gender, remoteness area, socio-economic index, and level of education) were entered. In the second stage, other non-focal variables (self-efficacy knowledge, self-efficacy coping, exposure to suicide, and a child’s history of service use, diagnosis and suicidal ideation) were entered; and finally, suicide stigma and literacy scores were entered in the third stage as the explanatory variables. The dependent variables were help-seeking attitudes and help-seeking intentions.

Sensitivity analyses re-estimated the parental help-seeking intention regression models for each source of help – that is, for general practitioner or doctor, school counsellor or nurse, clinical psychologist or mental health professional, psychiatrist, crisis support service, and emergency department.

## Results

Table 2 presents the mean, median, standard deviation and range, or frequency and percentage statistics for all variables, excluding the demographics already reported in Table 1.

**Table 2:** Descriptive Statistics for All Variables, Excluding Those in Table 1

	*N*	Frequency or *M*	Median	% or SD	Min.	Max.
Stigma subscale (SOSS-SF)	302	1.68	1.63	0.56	1	3.75
Isolation/depression subscale (SOSS-SF)	302	3.98	4	0.67	1.5	5
Glorification/normalization subscale (SOSS-SF)	302	2.53	2.5	0.77	1	5
Suicide literacy (LOSS-SF)	302	8.57	9	2.09	2	12
Help-seeking attitudes (ATSPPHS-SF)	302	27.45	28	2.75	16	30
Help-seeking intentions (GHSQ)	302	26.99	28	5.84	6	36
From a general practitioner/doctor	302	5.02	6	1.43	0	6
From a school counsellor/nurse	302	4.16	4	1.75	0	6
From a clinical psychologist/mental health professional	302	5.46	6	0.94	0	6
From a psychiatrist	302	4.27	4	1.73	0	6
From a crisis support service	302	4.56	5	1.53	0	6
From an emergency department	302	3.52	4	1.84	0	6
From none of those	302	0.25	0	0.73	0	6
Self-efficacy total (SE-SMHC)	302	73.02	75	12.67	27	90
Self-efficacy knowledge subscale (SE-SMHC)	302	40.33	42	8.05	10	50
Self-efficacy coping subscale (SE-SMHC)	302	32.69	34	5.98	13	40
Exposure to suicide	302	5.95	6	2.28	0	9
Child’s service use – in past twelve months^a^	302	196		64.90%		
From a general practitioner/doctor	302	161		53.31%		
From a school counsellor/nurse	302	87		28.81%		
From a clinical psychologist/mental health professional	302	164		54.30%		
From a psychiatrist	302	45		14.90%		
From a crisis support service	302	26		8.61%		
From an emergency department	302	36		11.92%		
From other	302	9		2.98%		
Child’s diagnosis – in past twelve months^a^	302	141		46.69%		
Anxiety disorders	302	122		40.40%		
Mood disorders	302	76		25.17%		
Personality disorders	302	8		2.65%		
Attention-Deficit/Hyperactivity Disorder / Oppositional Defiance Disorder / Conduct Disorder	302	31		10.26%		
Autism Spectrum Disorder	302	30		9.93%		
Eating disorders	302	5		1.65%		
Substance abuse disorders	302	6		1.99%		
Schizophrenia/psychosis	302	7		2.32%		
Other	302	3		0.99%		
Child’s suicidal ideation – in past twelve months^a^	302	144		47.68%		

*N* Number of responses, *M* Mean, *SD* Standard deviation

^a^These categories are not mutually exclusive, and some participants reported multiple answers. Further, parents had an average of two children each and the survey did not distinguish between which child (or children) received mental health care or diagnoses

### Stigma, Literacy & Help-Seeking Attitudes

Table 3 presents the results of the first hierarchical regression analysis assessing the effect of parental suicide stigma and literacy on help-seeking attitudes. At Stage 1, demographic variables did not contribute significantly to the regression model (*F*(5296) = 1.508, *p* = 0.187), accounting for only 2.5% of the variation in a parent’s help-seeking attitudes. Introducing the non-focal variables explained an additional 13.5% of variation in a parent’s help-seeking attitudes, and the overall model was significant (*F*(11,290) = 5.006, *p* < 0.001). This was attributable primarily to the role of the self-efficacy knowledge subscale (β = 0.30, *p* < 0.001). Finally, adding suicide stigma and literacy to the regression model explained an additional 2.8% of the variation in help-seeking attitudes, and the overall model was again significant (*F*(15,286) = 4.408, *p* < 0.001). Here, higher scores on the stigma subscale (β = −0.15, *p* = 0.012) and having a child with history of suicidal ideation (β = −0.14, *p* = 0.026) were associated with more negative help-seeking attitudes, while higher scores on the self-efficacy knowledge subscale continued to be associated with more positive help-seeking attitudes (β = 0.26, *p* < 0.001). No significant association was found between suicide literacy and help-seeking attitudes. The total amount of variance in parental help-seeking attitudes explained by the final model was 18.8%.

**Table 3:** Summary of the Hierarchical Regression Analysis for Variables Predicting a Parent’s Help-Seeking Attitudes (*N* = 302)

	Model 1		Model 2		Model 3	
Variable	*β*	Std. error	*β*	Std. error	*β*	Std. error
Age	0.002	0.027	0.010	0.026	0.002	0.027
Gender	0.084	0.575	0.085	0.557	0.071	0.561
Level of education	−0.095	0.332	−0.103	0.326	−0.117	0.336
Remoteness area	−0.041	0.288	−0.046	0.272	−0.054	0.273
IRSAD	0.095	0.066	0.032	0.064	0.021	0.063
Self-efficacy knowledge subscale			0.301**	0.024	0.256**	0.024
Self-efficacy coping subscale			0.071	0.032	0.059	0.032
Exposure to suicide			−0.082	0.073	−0.089	0.073
Child’s service use			0.072	0.391	0.058	0.390
Child’s diagnosis			0.057	0.369	0.047	0.366
Child’s suicidal ideation			−0.123	0.352	−0.144*	0.354
Stigma subscale					−0.150*	0.294
Isolation/depression subscale					0.002	0.224
Glorification/normalization subscale					−0.058	0.203
Suicide literacy					0.072	0.074
*R*^2^	0.025		0.160		0.188	
*F* for change in *R*^2^	1.508		7.749**		2.483*	

*p < 0.05. **p < 0.01

### Stigma, Literacy & Help-Seeking Intentions

Table 4 presents the results of the second hierarchical regression analysis assessing the effect of parental suicide stigma and literacy on help-seeking intentions. At Stage 1, demographic variables contributed significantly to the regression model (*F*(5,296) = 4.330, *p* < 0.01), accounting for 6.8% of the variation in a parent’s help-seeking intentions. Here, female gender (β = 0.15, *p* = 0.010) was positively associated and higher educational attainment (β = −0.19, *p* = 0.002) was negatively associated with help-seeking intentions. Introducing the non-focal variables explained an additional 5.8% of variation in a parent’s help-seeking intentions, and the overall model was significant (*F*(11,290) = 3.810, *p* < 0.001). Female gender (β = 0.18, *p* = 0.003) continued to be positively associated with help-seeking intentions, while higher educational attainment (β = −0.16, *p* = 0.007) and remote dwelling (β = −0.13, *p* = 0.041) were negatively associated with help-seeking intentions. At Stage 3, adding suicide stigma and suicide literacy to the regression model explained an additional 2.2% of the variation in help-seeking intentions, and the overall model was again significant (*F*(15,286) = 3.308, *p* < 0.001). Here, higher scores on the stigma subscale were associated with lower help-seeking intentions (β = −0.16, *p* = 0.010). Female gender (β = 0.16, *p* = 0.008) continued to be positively associated with help-seeking intentions, and higher educational attainment (β = −0.16, *p* = 0.010) and remote dwelling (β = −0.14, *p* = 0.025) were negatively associated with help-seeking intentions. Finally, parental help-seeking attitudes was added to the regression model, which explained an additional 15.7% of the variation in help-seeking intentions, and the overall model was again significant (*F*(16,285) = 7.821, *p* < 0.001). In the final model, parents with more positive help-seeking attitudes (β = 0.44, *p* < 0.001) and who were female (β = 0.13, *p* = 0.020) had significantly higher intentions to seek help, whereas those living in remote areas (β = −0.12, *p* = 0.039) had significantly lower intentions to seek help. Neither suicide stigma nor literacy were significantly associated with help-seeking intentions in the final model. The total amount of variance in help-seeking intentions explained by the final model was 30.5%.

**Table 4:** Summary of the Hierarchical Regression Analysis for Variables Predicting a Parent’s Help-Seeking Intentions (*N* = 302)

	Model 1		Model 2		Model 3		Model 4	
Variable	*β*	Std. error	*β*	Std. error	*β*	Std. error	*β*	Std. error
Age	−0.017	0.057	0.009	0.057	−0.017	0.059	−0.017	0.053
Gender	0.149*	1.195	0.177**	1.208	0.156**	1.221	0.125*	1.107
Level of education	−0.188**	0.689	−0.164**	0.707	−0.162*	0.731	−0.110	0.666
Remoteness area	−0.112	0.598	−0.127*	0.589	−0.141*	0.594	−0.117*	0.538
IRSAD	−0.006	0.137	−0.049	0.138	−0.061	0.138	−0.071	0.125
Self-efficacy knowledge subscale			0.130	0.052	0.091	0.053	−0.022	0.049
Self-efficacy coping subscale			0.125	0.070	0.112	0.070	0.086	0.064
Exposure to suicide			0.056	0.157	0.046	0.158	0.085	0.144
Child’s service use			0.053	0.848	0.041	0.849	0.016	0.769
Child’s diagnosis			0.018	0.799	0.013	0.797	−0.007	0.722
Child’s suicidal ideation			−0.084	0.763	−0.104	0.771	−0.040	0.703
Stigma subscale					−0.157*	0.640	−0.091	0.586
Isolation/depression subscale					−0.034	0.488	−0.034	0.441
Glorification/normalization subscale					−0.016	0.441	0.010	0.400
Suicide literacy					0.009	0.160	−0.023	0.145
Help-seeking attitudes							0.440**	0.116
*R*^2^	0.068		0.126		0.148		0.305	
*F* for change in *R*^2^	4.330**		3.215**		1.811		64.490**	

*p < 0.05, **p < 0.01

Sensitivity analyses at the item-level of the help-seeking intentions scale found that higher scores on the stigma subscale were associated with lower intentions to seek help from a school counsellor or nurse (β = −0.15, *p* = 0.020); and higher scores on the suicide literacy scale were associated with lower intentions to seek help from a psychiatrist (β = −0.16, *p* < 0.001).

## Discussion

Suicide is the leading cause of death among Australian young people, yet rates of help-seeking for suicidal ideation and behaviors in this population are concerningly low. Given parents are often key facilitators of their child’s access to professional mental health services, this study examined the relationships between parental suicide stigma, parental suicide literacy, and their attitudes and intentions toward seeking professional help for their child if they were to express thoughts of suicide. The study found evidence that lower parental suicide stigma was significantly associated with more positive help-seeking attitudes and higher intentions to seek help for one’s child. Parental suicide stigma was particularly associated with lower intentions to seek help from a school counsellor or nurse. Contrary to expectations, parental suicide literacy was not significantly associated with either help-seeking attitudes or intentions. However, parental suicide literacy was significantly associated with lower intentions to seek help from a psychiatrist, potentially stemming from the knowledge that general practitioners or psychologists are first-line treatment options.

### Theoretical Implications of the Findings

The finding that parental suicide stigma was associated with more negative help-seeking attitudes and lower help-seeking intentions is consistent with past research on personal help-seeking for *oneself*, which found that higher levels of suicide stigma were significantly associated with more negative attitudes toward help-seeking and lower intentions of seeking help in a community-based sample (Calear et al., 2014b). They also support previous findings regarding mental health stigma and help-seeking for general mental health problems (Barney et al., 2006; Reynders et al., 2014). The present study indicates that, just as individual suicide stigma can reduce the likelihood of seeking professional help for oneself, a parent’s suicide stigma reduces the likelihood of seeking professional help for one’s child. The association may be attributable to a number of factors, including a parent’s denial of or failure to recognize their child’s problems, a preference for self-reliance when solving them, or a fear of blame, stigmatization or negative feedback from family and friends (Barney et al., 2006; Zartaloudi & Madianos, 2010).

Conversely, the finding that higher parental suicide literacy was not associated with more positive help-seeking attitudes and higher help-seeking intentions is in contrast to past research on personal help-seeking, in which suicide literacy has been linked to more positive attitudes toward help-seeking and higher intentions of seeking help in a community-based sample (Calear et al., 2014b). It also contradicts previous findings into mental health literacy and help-seeking for general mental health problems, which found the same – that increased mental health literacy is associated with help-seeking (Bonabi et al., 2016). These differences may be attributable to the limited variability and relatively high levels of suicide literacy in the current sample. While the high level of suicide literacy in this community sample is a welcome finding, it may have produced a ceiling effect. For this reason, the non-significant results should be interpreted with caution.

There were a number of additional findings related to the non-focal predictor and demographic variables. First, higher scores on the self-efficacy knowledge subscale were associated with more positive help-seeking attitudes in parents. This finding is consistent with existing evidence that individuals who reported they would encourage others to seek help for emotional distress had significantly higher levels of self-efficacy compared to those who reported they would not encourage help-seeking in others (Moore et al., 2015). The finding is unsurprising, in that parents who felt most confident in their ability to access help and interact with the healthcare system were more likely to report positive attitudes about doing so. However, the strength of this relationship was unexpected. Once all independent variables were included in the model, the self-efficacy knowledge subscale was the most important predictor for a parent’s help-seeking attitudes. Thus, it will be important to explore this relationship further in future research.

There was an unexpected and worrying significant relationship between a child’s history of suicidal ideation and parents having more negative help-seeking attitudes. Past research has reported mixed findings on this issue (Carlton & Deane, 2000; Saunders et al., 1994). The current relationship can be considered in a number of ways – it may reflect help-negation and a sense of hopelessness or nihilism about the future; alternatively, it may be the outcome of prior negative help-seeking experiences (Deane et al., 2001). Or, parents may view their child with a history of suicidal ideation as seeking attention or being over-dramatic by disclosing thoughts of wanting to end their life, but never putting those goals and desires into action (Gregory, 2012). Hence, they may not take suicide risk seriously and negate seeking help for this reason (Gregory, 2012). Given the potentially tragic consequences of help-negation for suicidal individuals, there is a need for future research in this field, potentially using qualitative measures to explore parents’ reasons for not seeking help.

Mothers had significantly higher help-seeking intentions compared to fathers. The majority of past research indicates the same – that females have more positive help-seeking attitudes and higher help-seeking intentions (Calear et al., 2014b; Rickwood & Braithwaite, 1994). Further, females typically have less stigmatizing attitudes (Sheffield et al., 2004), which in turn may affect their help-seeking intentions. Again, this finding suggests that, just as gender affects one’s own likelihood to seek professional help, a parent’s gender may also affect their likelihood to seek professional help for their child. Alternatively, it may reflect the traditional primary caregiver role of women, on whom the burden of care for a distressed child most commonly falls (Oruche et al., 2012). It is important to note that females were highly over-represented (91%) in the current sample – providing further evidence that mothers were more likely to self-select for participation in a study on children’s mental health. This also means that the study was not well-powered to evaluate gender differences and so the finding should be interpreted with caution.

Living in regional and remote areas was associated with lower intentions to seek help for a child. This finding is consistent with past research that showed living in an urban area increased the likelihood of a family accessing mental health treatment (Cohen & Hesselbart, 1993). This finding is particularly significant in a country like Australia, where some rural areas are sparsely populated and have limited local services. The lower intentions to seek help may be explained by heightened levels of mental health and suicide stigma in regional and remote Australia. Stigma and discrimination are thought to be particularly problematic in smaller communities (Komiti et al., 2006), and are associated with lower help-seeking (Cohen & Hesselbart, 1993). Additionally, those living in regional and remote communities may have less access to professional mental health services (Rickwood, 2005). Therefore, they may hold fewer intentions to seek professional help because those services (psychiatrists, school counsellors, etc.) are unavailable to them. Thus, encouraging appropriate help-seeking behaviors by improving service access should be a priority for regional and remote Australian communities, with consideration of alternative care pathways including telehealth and digital programs. Again, it must be noted that the current sample lived predominately in urban areas. Thus, this finding should be interpreted with caution and investigated further with a larger sample of regional and remote participants.

### Strengths, Limitations and Directions for Future Research

Some strengths and limitations of the current study warrant consideration. Firstly, while the recruited sample was large and diverse, it was not representative of the broader Australian population. Although social media platforms, like *Facebook*, are far-reaching, certain groups, including older adults and individuals with lower education and socioeconomic status, tend to be underrepresented (Batterham, 2014; Cowling, 2019). Further, individuals who voluntarily respond to surveys tend to be more invested in the content of that survey than those who do not respond (Wijnen et al. 2007). In the current study, this was demonstrated by the high rate of parents of a child with a history of suicidal ideation in the sample (48%), relative to the general population (29.9%, Evans et al., 2005). Positively, these are the parents for whom help-seeking attitudes and intentions are most critical to youth suicide prevention. Nonetheless, although research has found that samples obtained via internet recruitment are similarly representative as those obtained via traditional techniques (Batterham, 2014), online sampling may constrain the internal validity and generalizability of results, so caution should be taken when applying them outside the sample population. Future research could validate this study’s findings by using more representative samples.

Caution should also be taken when drawing conclusions overall since help-seeking attitudes and intentions are imperfect predictors of actual help-seeking behavior. Moreover, the internal consistency of the help-seeking attitudes measure (ATSPPHS-SF) was low in this study (ɑ = 0.67), compared to the original (ɑ = 0.85; Fischer & Farina, 1995) – potentially due to it being adapted to ask about help-seeking for one’s child, rather than oneself, and covering a range of different attitudes related to help-seeking processes. In future, the scale items may be revised to yield a stronger measure of a parents’ attitudes toward seeking professional psychological help for their child. Prospective longitudinal research could also be used to develop and test formal models of help-seeking for suicide, by more accurately assessing the directionality and mediation of these relationships, and determining whether a parent does follow through with their reported attitudes and intentions by actually seeking help for their child reporting suicidal ideation or behaviors.

Finally, in both regression models, over half of the variance remained unexplained, suggesting that other unmeasured factors also contribute to a parent’s help-seeking attitudes and intentions. Though the focus of the current study was on modifiable factors, it would be useful to explore a broader range of variables in the future, including norms (Fishbein & Ajzen, 2011), previous experiences with services (Cunningham & Freiman, 1996; Zwaanswijk et al., 2003), access to services, and systemic barriers like wait-times and costs (Reardon et al., 2017).

### Practical Implications of the Findings

This research contributes to our understanding of youth suicide prevention by identifying key facilitators of, and barriers to, parents seeking help for their adolescent children. For youth suicide prevention to be effective, a comprehensive multi-sectoral strategy is required, with communities, and particularly parents, playing a critical role in providing social support to vulnerable adolescents, engaging in follow-up care, fighting stigma, and supporting those bereaved by suicide (World Health Organization, 2014). Until recently, parents have not been the focus or target of suicide prevention programs, despite their key role in supporting adolescents to access mental health services and support (Sayal, 2005; Zwaanswijk et al., 2003).

Given the results of the current study, it is imperative that parents have the opportunity to be trained as gatekeepers in future, so that they have the knowledge and skills to effectively support adolescents in distress and become a key part of youth suicide prevention activities. Such programs should center on reducing suicide stigma and improving self-efficacy among parents, to encourage more positive attitudes and higher intentions to seek professional help for their child if they were to express thoughts of suicide. Further, the programs could particularly target fathers, remote populations, and parents of a child with a history of suicidal ideation, as these individuals had the most negative help-seeking attitudes and lowest help-seeking intentions in our sample.

## Conclusion

Suicide is the leading cause of death among Australian young people, yet rates of help-seeking for suicidal ideation and behaviors in this population are concerningly low. Given parents are often key facilitators of their child’s access to and engagement with mental health services, this study examined the relationships between parental suicide stigma, parental suicide literacy, and their attitudes and intentions toward seeking professional help for their child if they were to express thoughts of suicide. Overall, results showed that lower parental suicide stigma was significantly associated with more positive help-seeking attitudes and higher intentions to seek help for one’s child. Parental suicide literacy was not significantly associated with either help-seeking attitudes or intentions. Additionally, higher parental self-efficacy and having a child without history of suicidal ideation were significantly associated with more positive help-seeking attitudes; and female gender, urban dwelling and positive help-seeking attitudes were significantly associated with higher intentions to seek help. This research adds to the body of youth suicide prevention literature by improving the understanding of help-facilitating behaviors in parents. By establishing some potentially modifiable factors that promote or prevent a parents’ likelihood to seek professional help for their child, it may now be possible to identify ways to improve rates of help-seeking and access to services by adolescents. In turn, more young people may reach the services they need when they need them, with the goal of ultimately reducing adolescent deaths by suicide in Australia.
